# Surgical method to prevent early death of neonatal rat pups with Hirschsprung disease, thus permitting development of long-term therapeutic approaches

**DOI:** 10.1093/biomethods/bpac004

**Published:** 2022-01-27

**Authors:** Lincon A Stamp, Enie Lei, Jamie J M Liew, Ruslan V Pustovit, Marlene M Hao, David H Croaker, John B Furness, Cameron D Adams

**Affiliations:** 1 Department of Anatomy and Physiology, University of Melbourne, Parkville, VIC 3010, Australia; 2 Florey Institute of Neuroscience and Mental Health, Parkville, VIC 3010, Australia; 3 Division of Paediatrics and Child Health, Canberra Hospital, Canberra, ACT 2605, Australia

**Keywords:** Hirschsprung disease, aganglionic colon, intestinal surgery, stoma formation, enteric nervous system

## Abstract

Hirschsprung disease occurs when children are born with no intrinsic nerve cells in varying lengths of the large intestine. In the most severe cases, neurons are also missing from the distal part of the small intestine. Nerve-mediated relaxation of the aganglionic bowel fails and fecal matter accumulates in the more proximal regions of the intestine. This is life threatening. Perforation of the bowel can ensue, causing sepsis and in some cases, death of the infant. Repopulation of the colon with neural stem cells is a potential therapy, but for this to be successful the patient or experimental animal needs to survive long enough for neural precursors to differentiate and make appropriate connections. We have developed a surgical procedure that can be applied to rats with Hirschsprung disease. A stoma was created to allow the normal bowel to empty and a second stoma leading to the aganglionic bowel was also created. This allowed homozygous mutants that would usually die at less than 3 weeks of age to survive into adulthood. During this time, the rats also required post-operative care of their stomas. The interventions we describe provide an animal model of Hirschsprung disease that is suited to assess the effectiveness of cell therapies in the treatment of this condition.

## Introduction

Hirschsprung disease (HSCR) is a congenital disorder characterized by the absence of nerve cells of the enteric nervous system (ENS), referred to as aganglionosis, in the distal regions of the gastrointestinal tract [1–3]. The lack of enteric neurons, including inhibitory neurons that relax the colon, results in absence of the reflex pathways required for colonic propulsion meaning patients are unable to pass fecal content. Existing surgical interventions involve the removal of the aganglionic region followed by an anastomosis of the healthy, ganglionated gut to the anus [4–6]. In cases where there is significant build-up of fecal content in the colon, the child is premature, or pull-through surgery is otherwise contraindicated, a temporary colostomy is performed before surgical intervention to remove the aganglionic region and re-anastomose the gut [7, 8]. While life-saving, these surgical interventions can often be followed by chronic complications such as fecal incontinence and constipation [8–12], which have been shown to result in a reduced quality of life for many HSCR patients [12–15]. This creates a need for other approaches to treatment.

One promising approach in the treatment of HSCR is the use of neural stem cells [16–20]. Previous work has shown that human-derived ENS lineages [21] and ENS precursors from mice [17] can recolonize the aganglionic regions in experimental animals and form networks that resemble the mature ENS. However, a significant limitation of these studies to date is that recipient mice do not live longer than 4–5 weeks due to bowel obstruction and enterocolitis [17, 22]. This period of time is not long enough for recolonization and restoration of function in long sections of colon, nor to demonstrate the long-term survival and function of newly generated neurons. Ideally, it would be advantageous to be able to bypass the aganglionic bowel, recolonize the region deficient of the ENS and once recolonization and function is restored, reestablish the continuity of the colon. However, the mouse is too small to readily carry out the surgery required to maintain animals while the stem cells develop and to later restore the continuity of the colon. Hence, we have utilized a rat model.

The spotting lethal (*sl/sl*) rat has a naturally occurring null mutation in the *EdnrB* gene [23, 24], a mutation which also occurs in some human patients resulting in Hirschsprung disease [25–29]. The deletion in rat spans the distal half of the first coding exon and the proximal part of the adjacent intron of *EdnrB* and results in a Hirschsprung disease phenotype in these rats [23, 24]. The aganglionosis involves a long segment that can extend from the distal ileum to the rectum or from the proximal colon to the rectum which creates a megacolon similar to that seen in HSCR patients. This length of aganglionic bowel makes them particularly useful for the investigation of stem cell therapy.

In this paper, we describe an optimized surgical procedure to bypass the aganglionic bowel and the necessary post-operative care to ensure the long-term survival of these animals. In doing so, we have extended the life of the animal which provides the opportunity to assess the effectiveness of cell therapy treatments in the aganglionic bowel to recolonize and restore function.

## Materials and methods

### Ethical statement

All procedures in this study were approved by the Animal Ethics Committee of the Florey Institute of Neuroscience and Mental Health (Ethics Number 19-004) and complied with the Australian Code for the Care and Use of Animals for Scientific Purposes (National Health and Medical Research Council of Australia). After weaning, animals were housed individually with environmental enrichment under a 12-h light–dark cycle with *ad libitum* access to food and water.

### Surgical preparation

Surgery is conducted on 7–10-day-old postnatal *EdnrB sl-/sl-* (KO) rat pups weighing between 6 and 10 g. We performed genotyping to distinguish KO pups from their heterozygous and wild-type littermates (using primers flanking the 301-bp deletion of the mutant *EdnrB* gene [23]) but KO pups can also be distinguished by their white coat and gray facial markings or spots. To facilitate the dam accepting the pups back after surgery, KO animals, together with some of their littermates, are placed into a small cardboard box (24 × 13 × 6 cm) lined with tissues and bedding from the home cage which is kept warm using a heat mat (37°C). Pups remain separated from the dam for the surgical period as well as the post-operative recovery from anaesthesia.

In preparation for surgery, pups are anaesthetized in a small animal induction chamber (19 × 13 × 6cm) circulated with 4.0–4.5% isoflurane vaporized in 1 L/min oxygen and are maintained at 1.5–2.0%. Pups are then placed onto a heated mat warmed to 37°C in a supine position by applying masking tape across the hind limbs. The abdomen is then cleaned with 80% ethanol and any fur removed using a straight edge razor blade, followed by a further clean with 80% ethanol.

### Stoma surgery

A video illustrating the surgical procedure (Supplementary Video S1) is included in the Supplementary Material.

To begin, an incision is created through the skin in the midline using fine scissors. The abdomen is then accessed by cutting through the abdominal wall along the linea alba (to minimize bleeding). The next step is to locate and externalize the cecum and proximal colon. To locate the cecum, it can help first to gently externalize the distal part of the small intestine using sterile cotton tips that are wet with sterile saline. Once the cecum is externalized, most of the small intestine can be returned to the abdominal cavity. A piece of suture can be used to identify the colon. To keep the externalized cecum and proximal colon, as well as the abdominal cavity moist, apply drops of sterile saline as needed.

Two openings for the stomas are then created through the skin and the abdominal muscle, approximately 3–5 mm lateral to the midline. The skin is gently lifted and an incision that exposes the abdominal muscle is made with sharp scissors. A region of the muscle wall is then pulled through the stoma opening and cut through using sharp scissors, so that the openings in the skin and abdominal wall muscle are aligned.

**Figure 1:**
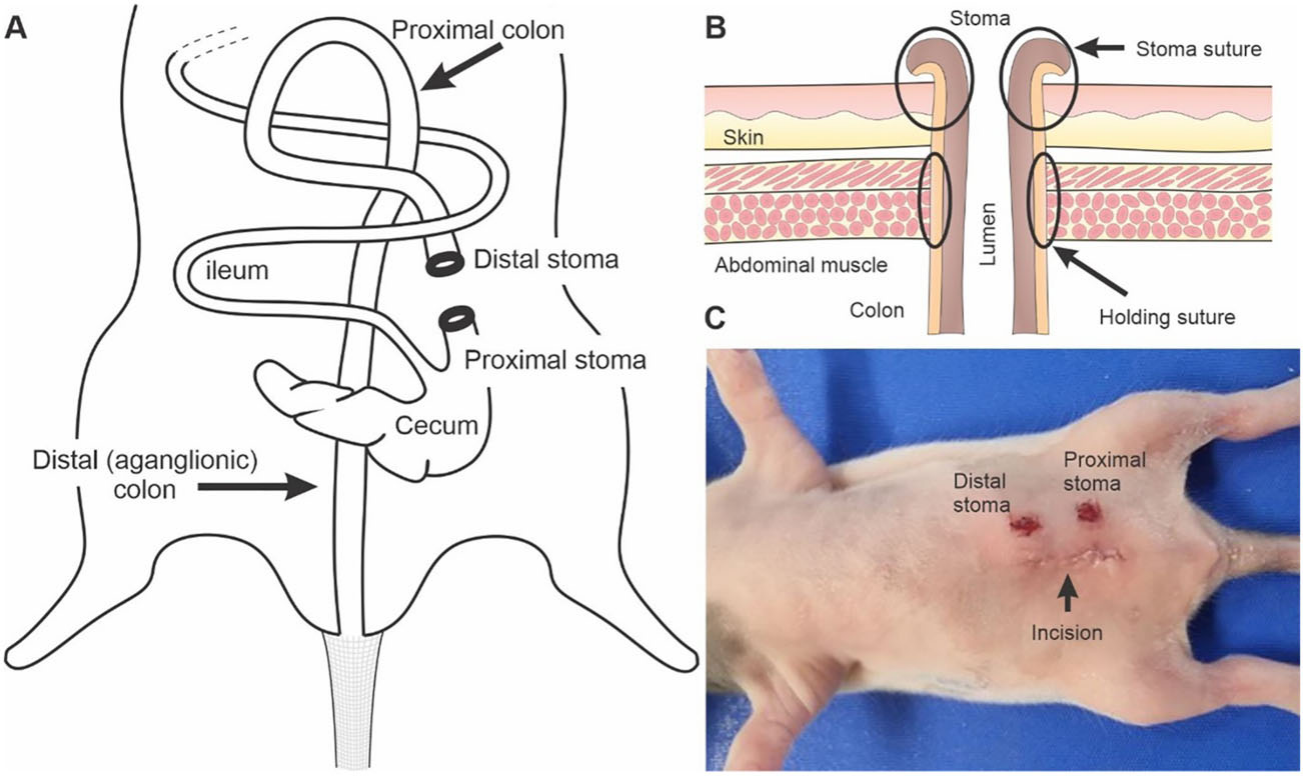
The stoma surgery. (**A**) The positions of the stomas and their relation to the rat gastrointestinal tract. The proximal stoma, distal to the caecum, is functional (passing content) whereas the distal stoma, connected to the colon, is non-functional (not passing content). (**B**) The cut end of the colon is drawn through aligned openings in the abdominal muscle and skin. It is held in place by two holding sutures connecting it to the abdominal muscle. Four individual stitches are used to join the opening of the colon to the skin, creating the stoma. (**C**) The appearance of the pup after the completion of surgery.

After identifying the agangalionic region, the proximal colon is then cut through (Fig. 1A). To do this, place the externalized proximal colon and caecum onto a gauze pad that is wet with sterile saline. The agangalionic region can be identified by the constriction (narrowing) of the colon and the absence of fecal content. Find a suitable place between the vascular arcades and cut through the colon, about 0.5 cm proximal to the constriction using sharp scissors. This is to ensure that the entire colon leading to the functional stoma is ganglionated. Remove any fecal content from the open ends of the severed colon with a wet cotton tip. This severed gut will create a functional part leading from the caecum (passing content) and the non-functional part leading to the anus (not passing content).

Next, guide the end of the non-functional gut leading to the anus toward and through the distal stoma opening. First, the cut end is drawn through the abdominal wall opening and anchored to the muscle (holding sutures, Fig. 1B) approximately 2–3 mm from its cut end using simple interrupted serosa–mucosa sutures (absorbable 8-0 USP poly-glycolic acid braided suture). When placing the sutures, it is important to minimize traumatizing the wall of the gastrointestinal tract. It is therefore better to avoid holding the wall of the gastrointestinal tract with grasping forceps. A tapered point needle, not a cutting needle is used. When the sutures are being placed, it is important to follow the curvature of the needle to reduce trauma to the soft tissue.

The colon is then sutured to the skin by passing sutures through all layers of the bowel wall using simple interrupted stitches (absorbable 8-0 USP poly-glycolic acid braided suture), ensuring that the lumen is visible and the stoma remains open (stoma sutures, Fig. 1B). The gut should be joined to the skin in at least four locations, equally spaced around the circumference of the stoma. When suturing the gut to the muscle wall or to the skin, tearing of the gut is possible if the sutures are too tight. The stitches should firmly hold the skin and abdominal wall together without cutting into the tissue.

The functional gut leading from the caecum to the proximal stoma is then connected using the same technique. After, the midline abdominal wall incision is closed as a single layer with a simple continuous suture (absorbable 8-0 USP poly-glycolic acid braided suture) followed by the skin with intradermal sutures (absorbable 6-0 USP poly-glycolic acid braided suture) (Fig. 1C).

Note that the rotation of the gut during development means that the more rostral stoma is distal with respect to the gut and the more caudal stoma is proximal with respect to the gut (Fig. 1A).

### Post-surgical care

A video illustrating animal behavior post-surgical rescue (Supplementary Video S2) is included in the Supplemental Material.

After the completion of surgery, the operated pup is injected with Carprofen (5 mg/kg, subcutaneous) and placed back into the cardboard box containing its littermates. It is important that a new cardboard box is used for each litter. The operated pup needs to be monitored until it is sufficiently responsive. After the completion of all required surgeries and when all operated pups show similar mobility to their littermates, all pups are placed back into their home cage with the dam. The operated pups need to be observed for 15–30 min to monitor for rejection from the dam. If this occurs, pups can be placed with a foster dam who has pups of a similar age (±2 days of the rejected pup). If no foster dam is available, the pup is euthanized.

Pups need to be monitored twice daily. It is important to ensure that the stomas remain open and are clean by shaving the area of the ventral abdomen around the stomas every 5 days. In the event of dermatitis from content irritation around the stoma or hind paws, we apply the topical cream Neocort (20 mg/g lignocaine, 5 mg/g neomycin, and 5 mg/g hydrocortisone). In severe cases, where a significant proportion of the abdomen, more than 5 mm wide, is red and irritated, we treat pups with the injectable NSAID Meloxicam (2 mg/kg, once daily, subcutaneous) and the injectable antibiotic Baytril (enrofloxacin, 5 mg/kg, once daily, subcutaneous) for 3–5 days.

It is also helpful to regularly irrigate the non-functional gut at least twice a week to prevent excessive mucus build up. To do this, we gently massage the mucus out of the distal stoma, or if required, through manual irrigation. The colon is first lubricated with a saline enema using a 1-mL syringe and atraumatic cannula (1–3 mL of saline depending on the size of the animal). Saline is then gently pushed though the distal stoma to flush mucus out through the anus (saline is continually flushed until the mucus is completely removed).

Starting from 3 weeks of age, we also provide an oral rehydration and energy solution (ORES) drinking bottle alongside water drinking bottles (both bottles provided *ad libitum*). This solution consists of 3.5 g NaCl, 1.5 g KCl, 2.9 g tri-sodium citrate, and 20 g D-glucose in 1 L of distilled water which help weight gain and replace fluid lost with the stool.

We have found that operated pups can be weaned at 4 weeks of age when they weigh more than 35 g. We use fiber cycle cat litter for bedding and provide four paper towels for nesting materials. Paper towels should be changed daily while bedding should be changed twice a week.

## Results and discussion

In this study, 136 animals underwent surgery using this technique with 131 recovering and returned to the home cage. In the pilot, 56 pups were operated on while in the main study 80 pups were operated on. As it stands, we have taken rats to 21 weeks of age (20 weeks after rescue surgery) before euthanizing them for investigation. This is well past the 1–5 week life expectancy of the *sl*/*sl* rat as first described for this strain by Ikadai and colleagues [30]. In our hands, survival of KO pups until weaning at 3 weeks post-surgery was >90%. Due to their small size, it was not practical to take biopsies to confirm aganglionosis. However, investigation of the colons from rescued animals using immunohistochemistry for the enteric neuron marker Hu confirmed that the colons were aganglionic (unpublished data).

In pilot experiments, the surgically rescued pups failed to thrive and we suspected they were receiving inadequate nutrition. Rodents, like many species, obtain nutrients through the bacterial production of short-chain fatty acids in the colon and also utilize vitamin B of bacterial origin [31]. Furthermore, content emerging from the functional stoma was very watery, potentially due to the lack of colonic re-absorption of water and electrolytes. To overcome the potential deficiencies related to the lack of short-chain fatty acids from the colon as well as the loss of water and electrolytes, we provided the rescued rats with an ORES that contained both electrolytes and glucose as an energy substrate. This addition resulted in improvements to animal well-being including weight gain and changes to the level of watery content produced. If a rat still grew slower than its littermates (in this study *n* = 5), we provided commercial puppy milk and/or a mash composed of rat pellets and milk until the pup was the same size as its littermates.

We have also found that removal and return of the non-surgery littermates (heterozygous and wild-type pups) at the same time as the surgery pups has reduced maternal rejection and improved survival rates. Minimizing foreign smells and tastes, by including some of the bedding from the home cage in the post-surgery cardboard box, has also reduced maternal rejection and loss of animals.

Animals were weaned 3 weeks post-surgery and we often observed soiling and irritation of the skin around the stoma and the lower limbs. We noted what seemed to be a behavioral adaptation of the rats in that they adopted a wide hindlimb stance (see Supplementary Video S2) which appeared to help avoid soiling and irritation of the hindlimbs. We also observed the rats cleaning the stoma region by licking. Regular shaving around the stomas also prevented content from getting stuck in the fur and irritating the skin. This is especially important after weaning when the pups are still learning to take care of themselves.

Another addition to our post-surgical care regime, which improved long-term survival rates, was the irrigation of the colon. We found it was common for the distal (non-functional) stoma to close over and the tissue to adhere and maintain the closure. By delivering saline through the distal stoma opening with an atraumatic cannula (with the saline flowing out the anus), we minimized the likelihood of the stoma closing. We also found irrigation of the colon critical to post-surgical care as it also prevented mucus build up in the lumen. If the non-functional colon is not irrigated every 2–3 days, the buildup will gradually become more viscous and difficult to dislodge.

While the surgical intervention and post-surgical care described here significantly increased life expectancy and animal well-being, we see two obvious issues that could arise with attempting to restore function using neural stem cells. The first is that the aganglionic colon may not be a viable host tissue. However, published work in mice indicates that neural progenitors are capable of generating both neurons and glia when they are transplanted into the aganglionic colon [17]. Second, there may be atrophy of the muscle due to disuse, which may not permit movements to be restored. In light of this, we have investigated the tissue histologically using hematoxylin and eosin staining and found that all layers were intact and had a normal appearance with no fibrosis evident. In future, we plan a detailed investigation of all tissue components in the rescued rats, compared with wild-types. As it stands, the next challenge will be re-anastomosis of the colon following re-colonization with enteric neurons.

Here, we have described a surgery and a regime of care that allows Hirschsprung disease rat pups to survive in a healthy state for over 5 months. This is advantageous, as studies performed in mice do not survive past 4–5 weeks [17, 22] which makes it difficult to investigate the effectiveness of implanted neural stem cells to colonize the aganglionic bowel and restore colorectal function. Moreover, it also provides an experimental platform to study other deficits that may occur in Hirschsprung disease and to investigate other ways to treat the disorder.

## Supplementary data

Supplementary data are available at *Biology Methods and Protocols* online.

## Data availability

The data that support the findings of this study are available from the corresponding authors, J.B.F. and C.D.A., upon reasonable request.

## Author contributions

L.A.S. and J.B.F. designed the experiments. D.H.C. originated the colony. L.A.S., E.L., J.J.M.L., R.V.P., and C.D.A performed the experiments. All authors contributed to writing and editing the manuscript. J.B.F. supervised all aspects of the work.

## Funding

This study has received funding from Takeda Pharmaceuticals and the Medical Research Future Fund (grant APP2009049). M.M.H. is a DECRA Fellow of the Australian Research Council.

## Disclosures

The authors’ laboratory receives funding from Takeda Pharmaceuticals for investigations of animal models of Hirschsprung Disease.

*Conflict of interest statement*. None declared.

## Supplementary Material

bpac004_Supplementary_Data
